# Caregiver Supervision Practices and Risk of Childhood Unintentional Injury Mortality in Bangladesh

**DOI:** 10.3390/ijerph14050515

**Published:** 2017-05-11

**Authors:** Khaula Khatlani, Olakunle Alonge, Aminur Rahman, Dewan Md. Emdadul Hoque, Al-Amin Bhuiyan, Priyanka Agrawal, Fazlur Rahman

**Affiliations:** 1 Johns Hopkins International Injury Research Unit, Department of International Health, Johns Hopkins Bloomberg School of Public Health, Baltimore, MD 21205, USA; khaulakhatlani@gmail.com (K.K.); pagrawa6@jhu.edu (P.A.); 2 Department of Preventive Medicine, Griffin Hospital, Derby, CT 06418, USA; 3 Centre for Injury Prevention and Research, Bangladesh (CIPRB), Dhaka 1206, Bangladesh; aminur@ciprb.org (A.R.); al_amin_prime@yahoo.com (A.-A.B.); fazlur@ciprb.org (F.R.); 4 International Centre for Diarrheal Diseases Research in Bangladesh (ICDDR,B), Dhaka 1212, Bangladesh; emdad@icddrb.org

**Keywords:** childhood unintentional injuries, drowning, drowning mortality, caregiver supervision, children under five, developing country, Bangladesh

## Abstract

Unintentional injury-related mortality rate, including drowning among children under five, is disproportionately higher in low- and middle-income countries. The evidence links lapse of supervision with childhood unintentional injury deaths. We determined the relationship between caregiver supervision and unintentional injury mortality among children under five in rural Bangladesh. We conducted a nested, matched, case-control study within the cohort of a large-scale drowning prevention project in Bangladesh, “SOLID—Saving of Children’s Lives from Drowning”. From the baseline survey of the project, 126 cases (children under five with unintentional injury deaths) and 378 controls (alive children under five) were selected at case-control ratio of 1:3 and individually matched on neighborhood. The association between adult caregiver supervision and fatal injuries among children under five was determined in a multivariable conditional logistic regression analysis, and reported as adjusted matched odds ratio (MOR) with 95% confidence intervals (CIs). Children under five experiencing death due to unintentional injuries, including drowning, had 3.3 times increased odds of being unsupervised as compared with alive children (MOR = 3.3, 95% CI: 1.6–7.0), while adjusting for children’s sex, age, socioeconomic index, and adult caregivers’ age, education, occupation, and marital status. These findings are concerning and call for concerted, multi-sectoral efforts to design community-level prevention strategies. Public awareness and promotion of appropriate adult supervision strategies are needed.

## 1. Introduction

Unintentional injuries among children are a major public health concern and a cause of substantial childhood morbidity and mortality [1]. The leading mechanisms of unintentional injuries among children less than 15 years are drowning, road traffic injuries, burns, and falls [2]. Among children aged 5 years and below, drowning is the leading cause of injury death globally, and over 90% of these deaths occur in low- and middle-income countries (LMICs) [3,4]. In Bangladesh, drowning accounts for 43% of all deaths in children aged 1–4 years. A child drowns every 30 min in Bangladesh, and 80% of these deaths occur within or around the home environment [3,4,5]. 

Whereas multiple factors contribute to the occurrence of an injury event among children under five years of age, lack of appropriate supervision is, however, considered to be a major contributing factor [6]. Supervision, in general, could be summarized by domains of attentiveness, proximity, and continuity, and can be considered as “the interaction of attentional behaviors and physical proximity extended in time” [1,6]. There is neither a consensus on the definition of supervision in the literature, nor on the quality of supervision required for preventing childhood unintentional injuries [7]. Indeed, distinctive environmental and behavioral factors shape parental attributes and parenting styles affecting the quality of supervision and occurrence of childhood unintentional injuries [8,9]. The lack of a standardized validated instrument tailored especially to the sociocultural contexts in LMICs further makes it difficult to explore supervision [6]. 

The evidence linking lapse of adult supervision with childhood unintentional injury deaths is mainly based on descriptive data from high-income countries (HICs), and few if any have assessed this relationship in a LMIC setting [10,11,12,13,14,15,16,17,18,19]. Hence, the objective of our study is to determine the relationship between adult caregiver supervision and unintentional injury deaths among children aged five years and below in rural Bangladesh. We hope that our study will contribute to the understanding of the role of supervision in preventing childhood unintentional injuries in LMICs, and to the design of effective childhood injury prevention strategies.

## 2. Materials and Methods

### 2.1. Study Design, Setting and Participants

We conducted a matched case-control study nested within the “SOLID—Saving of Children’s Lives from Drowning” project, which is a large-scale drowning prevention project in Bangladesh [20]. One of the aims of the SOLID project was to describe the burden and risk factors of drowning, and other injuries, among young children in Bangladesh. The SOLID project included a baseline census conducted in 51 unions (out of 83 unions) from seven sub-districts of rural Bangladesh: Matlab North, Matlab South, Daudkandi, Chandpur Sadar, Raiganj, Sherpur Sadar, and Manohardi. The census collected sociodemographic and injury outcome data on approximately 1.2 million people from 270,387 households.

The cases selected for our matched case-control study were children under five (male and female) that died from unintentional injuries in the SOLID census area, and the controls were similarly aged children who were alive over the same period. To ensure homogeneity of household, environmental, and socioeconomic characteristics between cases and controls, we selected controls from a neighborhood as close to their respective cases as possible. Controls were selected from the same Bari but a different household as that of the cases. A Bari is a collection of 2–10 households living in close proximity and within the same geographical compound; these households share resources including housing, water, food, and space for animals. If no suitable controls were found from a different household within the same Bari as a case, then the controls were selected from the same Bari and the same household or from an adjacent Bari within the same village.

Three controls were selected for every case to yield a matching ratio of 3:1. Our case-control study included all 126 cases of children under five who died due to unintentional injuries in the last 12 months prior to the census. In all, the sample size for our matched case-control study was 504 (126 cases and 378 controls). This sample size has more than 90% power to detect an odds ratio of 2 in mortality due to unintentional injuries, comparing cases to control, and assuming that the prevalence of supervision is 50% in the general population.

### 2.2. Study Variables and Study Tool

Unintentional injury was operationally defined as any household member who sought treatment or lost at least one working day or could not go to the school for at least one day due to an injury event [20]. The injury events listed included suicide, transport injury, violence, fall, cut injury, burn, drowning, poisoning, machine injury, electrocution, animal bite injury, injury by blunt object, suffocation, and others. We further categorized all injuries based on intent, and described them as: unintentional injuries, self-harm, and violence [21]. Drowning was defined as “the process of experiencing respiratory impairment from submersion/immersion in liquid” [22].

Death from unintentional injuries was the main outcome variable, and this data was collected over a one-year recall period. Supervision was the main exposure variable. Since physical proximity between a child and an adult caregiver is most closely correlated with supervision, we used physical proximity as a proxy indicator for supervision in our study [1,23]. Based on prior injury literature in children, a child was considered in proximity of the adult caregiver if both the child and the adult caregiver were at the same location, either inside or outside of the home during the review period [24,25]. We described specific locations inside and outside of a typical home in rural Bangladesh such that the co-location of both the child and adult caregiver at the same location would put the adult caregiver at arm’s length of the child. To avoid any social desirability bias, we divided a day into different periods and independently assessed the specific location of the child and adult caregiver at each of those periods. In this study, we only considered supervision during a review period between 9 am and 1 pm, as the majority of unintentional injury deaths (mainly drowning) in Bangladesh are reported to take place during this time period [5]. 

We defined an adult caregiver as the head of household or any adult 18 years of age or older who had primary responsibility for overseeing the child’s welfare during the period under review. While we recognized that older children at times could “supervise” younger children, the injury literature suggests that supervision by an adult may be more effective compared to supervision by an older child (especially an older child below 13 years of age) [26]. Supervision conducted by children younger than 13 years could in fact increase injury risk for the child that is being supervised [26]. 

We obtained all information using questionnaires designed based on guidelines for injury surveillance by the World Health Organization (WHO) [27]. The questionnaires were used to collect data on injury mortality and morbidity, sociodemographic characteristics, environmental factors, supervision practices, and drowning related factors and prevention practices for households with children under five [3,20].

### 2.3. Data Collection Procedure

Two sets of trained data collectors administered the questionnaires. Data were obtained from either the head of the household or any adult 18 years and older who was present in the house during face-to-face interviews. The first set of data collectors gathered information on household characteristics, birth history, household environment, and death confirmation. If an injury was reported, the second set of data collectors visited the household to collect data on injury morbidity, injury mortality, and mechanism of injury. The data collectors were adults 18 years and older who had completed at least secondary school education, and had undergone 15 days of field training. The field training included modules on the questionnaires, operational manual, survey implementation, research ethics, and data management. An initial pilot-testing of the questionnaires revealed a high percentage of agreement (>80%) regarding the data collected from the same households by different data collectors. The activities of the data collectors were supervised by the field supervisors, who reported directly to a field research or sub-district manager, and the managers reported directly to the central office. Daily collation of data was done at the union level with oversight from the field supervisor, after which data were transmitted to the sub-district level for preliminary cleaning before transmission to the central office. All questionnaires were translated from English to Bangla (Bangladesh’s local language), and were back-translated and pre-tested in the field. Ethical clearance was obtained from the Institutional Review Boards at Johns Hopkins Bloomberg School of Public Health (JHSPH), International Centre for Diarrheal Disease Research, Bangladesh (ICDDR,B) and Centre for Injury Prevention Research Bangladesh (CIPRB) (Ethical Approval Code 00004746). The study was conducted in accordance with the Declaration of Helsinki.

### 2.4. Statistical Analysis

First, we obtained data on adult caregivers’ sociodemographic characteristics for the selected cases and controls and linked these with the supervision and injury mortality data from the SoLiD baseline census. Second, we described the frequencies and percentages of all included variables. Third, we computed the unadjusted matched odds ratio (MOR) of unintentional injury deaths among children under five with 95% confidence intervals (CIs) using simple conditional logistic regression. Fourth, we described a multivariable model for the odds of unintentional injury deaths among children under five with adult caregiver supervision as the main explanatory variable, using a multiple conditional logistic regression. We added other predictors to the multivariate model, one at a time, checking the model significance each time based on the likelihood ratio test (*p* < 0.05). Last, we reported the adjusted MOR with 95% CI for adult caregiver supervision and other predictors from the multivariate model. 

To see if the odds ratios in our study approached the relative risk, we tested the rare disease assumption by assessing the prevalence of unintentional injury-related mortality among children under five from our source population. The characteristics of the selected controls and all living children under five from our source population were also compared to check if the controls were truly representative of our source population. We conducted all statistical analyses using STATA version 14 (StataCorp. 2015. Stata Statistical Software: Release 14. StataCorp LP, College Station, TX, USA).

## 3. Results

The SOLID baseline census database had a total of 112,664 children under five; 126 injury deaths were reported among these children over a one-year recall period. All injury deaths were unintentional except for one case that was undetermined. All unintentional injury deaths resulted from drowning except for 14 (11%) cases. Of the 14 cases, 5 (4%) were road traffic injuries, 4 (3.2%) suffocation, 2 (1.6%) cut injury, and 1 (0.8%) death each due to unintentional poisoning and animal bite injury. Three hundred seventy-eight (378) controls were selected at a 1:3 case-control ratio and matched on geographical location/neighborhood.

### 3.1. Baseline Characteristics of Children under Five and Their Caregivers

Overall, 18.4% of children were supervised (cases 8.7%; control 21.7%) (Table 1 and Table 2). A higher proportion of cases were males (cases 53.2%; controls 48.7%), and in the 1–4 years age group (cases 94.4%; controls 81.2%) (Table 2). The minimum age of the adult caregivers in our study was 18 years; overall 83.3% of the adult caregivers were in the age category 25–64 years (Table 3). Only 4.6% of children in the study had adult caregivers with education beyond secondary level (cases 3.2%; controls 5%). In Bangladesh, grades 1–5 are considered primary education, grades 6–12 secondary, and advanced level is grades 13 and above. The most common occupation among adult caregivers was agriculture/farming (28.6%), followed by those who were retired/unemployed/housewives (21%). A greater percentage of adult caregivers of cases were older than 65 years (cases 20.6%; controls 10.6%) and were transport workers (cases 11.1%; controls 6.1%). On the other hand, more adult caregivers of controls were traders as compared to cases (cases 13.5%; controls 20.6%) (Table 3). Both the cases and the controls were similar in terms of socioeconomic index as well as adult caregivers’ education and marital status. 

### 3.2. Univariate and Multivariable Analysis 

Based on the univariate model, supervision (unsupervised child compared to supervised), child’s age (1–4 years compared to those <1 year), adult caregiver’s age (≥65 years compared to those 24–65 years), and adult caregiver’s occupation (transport workers compared to traders) were significantly associated with unintentional injury-related mortality among children under five (Table 4). Based on the final multivariable model, children under five who died from unintentional injuries had 3.3 times increased odds of being unsupervised as compared to alive children (MOR = 3.3, 95% CI: 1.6–7.0), while adjusting for children’s sex (male, female), age (<1, 1–4 years), socioeconomic index (lowest, low, middle, high, highest), and adult caregivers’ age (18–24, 25–64, ≥65 years), education (none, secondary, A levels and above), occupation (trading, agriculture, skilled labor, unskilled labor, transport worker, student, retired/unemployed/housewife), and marital status (married, never married/divorced/widowed) (Table 4). In the adjusted analysis, likelihood of dying due to unintentional injuries also increased significantly if the child was aged 1–4 years as opposed to less than 1 year of age (OR = 5.2, 95% CI: 2.2–12.2), had an adult caregiver aged 65 years and above compared to age 25–64 years, (OR = 3.4, 95% CI: 1.5–7.6), and whose adult caregiver was a transport worker compared to a trader (OR = 3.4, 95% CI: 1.3–9.0). A child’s sex and socioeconomic status, and adult caregiver’s education and marital status were not significantly associated with unintentional injury-related mortality (Table 4). 

### 3.3. Comparison of Controls with the Source Population

The prevalence of unintentional injury was very low in our source data—126 per 112,664 total population or 0.1%. A comparison of the controls with other children under five from our source data did not yield any significant difference with respect to supervision status, age, sex, and socioeconomic status (Tables S1 and S2). Hence, we concluded that the rare disease assumption is reasonable given our data. 

## 4. Discussion

To our knowledge, our study is the first epidemiological study to have explicitly explored the relationship between adult supervision and unintentional injury deaths among children under five years of age in a rural setting of a developing country. We highlighted the protective role of adult supervision on fatal unintentional injuries of young children, especially drowning in rural Bangladesh. Our study suggested that the risk of unintentional injury mortality (mainly drowning) was three times higher among children younger than five years when they were not in close proximity of an adult caregiver, adjusting for covariates.

As far as the protective role of adult supervision in childhood unintentional injury prevention in an aquatic environment is concerned, our findings validate earlier studies [10,11,15,16,18,19,23,28,29]. While our findings are alarming, they are of public health significance. Once drowning occurs in a young child, death is almost certain and rapid. Even in instances where a child receives timely and adequate cardiopulmonary resuscitation, that child could still be faced with substantial morbidity such as neurological impairment [16]. Rural Bangladesh is mainly an agrarian society in which people build homes in close proximity to water bodies to have easy access to water for farming, bathing, cleaning, washing clothes/dishes, and recreational activities [5]. While our study highlights drowning as a main external cause of unintentional injury death among children under five (and the contribution of lack of supervision given Bangladeshi setting), lack of supervision is able to contribute to other leading external causes of injury in other settings depending on the environmental factors in those settings. 

Even though safety barriers between residences and water bodies can be an effective environmental intervention for drowning, it is nearly impossible to fence large natural water bodies, as in the case of Bangladesh [30,31,32]. Young children can experience drowning due to lapses in adult supervision despite physical barriers between the children and the water bodies [10,16]. Moreover, children under five who have the highest risk of dying due to drowning are seldom proficient swimmers [33,34]. The notion that giving swimming lessons to young children could reduce drowning deaths lacks sufficient evidence; in fact, there is recommendation against this strategy for children less than two years of age [31]. Therefore, primary prevention in the form of adequate adult supervision cannot be overemphasized in preventing childhood drowning deaths [23,31,32].

While the importance of attentiveness and continuity—two other pillars of the hierarchical model of supervision—cannot be overlooked, our results suggest that proximity could be used as a reasonable indicator of supervision in a resource-poor setting [6]. Physical proximity of the adult caregiver is regarded as the most vital component of supervision because it significantly diminishes risk-taking behavior among children [1,23,35]. This is crucial, as the assessment of supervision is most often difficult and subjective, especially in the absence of a standard tool. Morrengiello and House designed the Parents Supervision Attributes Profile Questionnaire in order to measure parental attributes which could indirectly predict parental supervision behavior and subsequent child risk-taking behavior [35]. However, it is not yet determined if the questionnaire is applicable in other sociocultural environments with different structural factors or in LMIC settings. It is noteworthy that the sociocultural environment of a community influences parental perceptions and cognitive behavior, which in turn affects the kind and level of supervision deemed appropriate for children [7]. The sociocultural differences that predispose children to greater injury risks have, however, not been established in the literature. To this end, future studies to develop supervision tools adapted for various settings and to examine the relationship between sociocultural differences in supervision and child injury risk would be desirable. 

Our results indicate that children aged 1–4 years are at significantly higher risk of unintentional injury mortality (mostly drowning), which is consistent with other studies [5,11]. Children aged 1–4 years are mobile, and they exhibit a higher risk-taking behavior than infants; thus, they are more exposed to the risk of unintentional injuries and require closer supervision [16]. As children get older, parents tend to underrate close supervision or misperceive children as efficient enough to deal with the injury risk themselves [36]. Current guidelines for child injury prevention should be explicit on the need to educate adult caregivers about providing continuous supervision to young children in hazardous environments [34,36]. Injury prevention programs, including drowning prevention programs, need to educate adult caregivers about children’s lack of motor skills required for self-rescue in event of an injury or near-submersion [11,36]. An adult caregiver’s knowledge of the child’s development has been shown to influence quality of supervision [37].

The finding of adult caregivers 65 years of age and older as a significant risk factor for childhood unintentional injury mortality is unique. Potential mechanisms could be elderly caregivers being limited physically to keep a close eye on the children, or intervene in the event of submersion in water. Moreover, elderly caregivers, such as grandparents, face considerable stress, exhaustion, and poor physical health and mental health conditions, which may impair them in being able to provide adequate or continuous supervision to young children [38,39,40]. Scarce data have, however, examined the role of supervision by elderly caregivers and child unintentional injury mortality, and the mechanisms through which more or less frequent injuries may occur under such observation. A study suggested protective, though statistically insignificant effect of grandparents’ supervision on medically-attended childhood injuries [41]. In contrast, our study focused on unintentional injury deaths, the majority of which were due to drowning, requiring caregivers to be physically active, vigilant, and close enough to young children to intervene and prevent such an incident from happening. However, adult caregivers above 65 years may not be able to do so due to their functional limitations. This calls for the promotion of low-cost and community-based programs that support child supervision by trained adults in LMICs, such as crèches (day care centers) [42]. Such programs have been demonstrated to reduce drowning and other unintentional injuries among children through the provision of close adult supervision in a safe environment [42]. The crèches allow working adults to perform their jobs, while similarly aged adults (crèche mothers) supervise children. 

As expected, male children were more likely to die from unintentional injuries than females [5,10,11]. Male children demonstrate higher risk-taking behavior while parents tend to supervise female children more closely [25,43]. It should be noted that unintentional injuries among children are a complex interplay of a child’s characteristics, environmental factors, parental beliefs, and supervision practices [25]. Consistent with prior studies, our study suggests that the risk of unintentional injury-related mortality decreased linearly with increasing level of adult caregiver’s education, though the results were not statistically significant [5]. Our finding of a significantly increased risk of child unintentional injury death among children supervised by adult caregivers who are transport workers compared with traders is remarkable and worthy of further inquiry, especially given that this association is independent of socioeconomic status. It would be interesting to explore if fatal injury events among children occurred at the same time and place where adult caregivers performed their jobs as transport workers. This information would be especially critical, in terms of childhood drowning and adult caregivers involved in water transport; however, lack of data prevented us from evaluating this further in our current study.

Our study is the first large population-based study in a LMIC to assess contribution of lack of adult supervision towards unintentional injury-related deaths among children under five. Using the rare disease assumption, our odds ratios could be used as a good approximation of relative risk, further strengthening the evidence we present in this study. We addressed selection bias by using a nested matched case-control study design and by demonstrating through analysis that the controls were truly representative of the source population [44] (Tables S1 and S2). 

Our study is not without limitations. First, we did not assess other domains of supervision— attentiveness and continuity—in this study due to lack of data. Based on prior evidence, we used physical proximity of the child with the adult caregiver as a proxy and assumed that having a young child within an adult caregiver’s reach enabled the adult caregiver to watch/listen to the child (attentiveness) without considerable interruption (continuity) [23]. Second, the cut-off age of 18 years that we had used for defining an adult caregiver may have excluded appropriate supervision by older children (especially those older than 13 years). We, however, explored this and did not find that the cut-off age differentially affected the cases compared to the controls. Hence, we maintained our conclusion about the relationship between adult supervision and child unintentional injury deaths. However, our study findings may not be generalizable to populations where supervision is provided mainly by older children or by multiple caregivers.

Third, we also did not include all of the alive children under five population as the comparator, but we did demonstrate that the selected controls were a true representation of the source population. Fourth, our model omits other important predictors of childhood unintentional injury such as, household size, adult caregivers’ substance abuse, beliefs, and personality, and children’s attributes including risk taking behavior and temperament due to lack of data. Fifth, to avoid losing power, we did not conduct sensitivity analysis based on the child’s age to account for the differences in supervision practices with respect to mobility of children. However, as the issue equally affects both cases and controls, we can maintain our conclusion about the relationship between adult supervision and child unintentional injury deaths. Last, it should be noted that the most common cause of unintentional injury mortality could be different if the study were to be replicated in another developing country, with an entirely different landscape than Bangladesh [45].

Our study is generalizable to other rural LMIC settings with a profile similar to rural Bangladesh. We advocate for approaches for enhancing adequate adult supervision of young children to decrease child injury risks, including drowning, in LMICs. 

## 5. Conclusions

Children under five years of age who died from unintentional injury-related deaths had a 3-fold higher likelihood of being unsupervised by an adult as compared to similarly aged living children, after matching for neighborhood factors and adjusting for children’s sex, age, socioeconomic status, and adult caregivers’ age, education, occupation, and marital status. Children’s age, and adult caregiver’s age and occupation were other significant predictors of deaths from unintentional injuries among these children. These findings are concerning and call for concerted, multi-sectoral efforts to deliver effective community-level prevention strategies. Public awareness and promotion of appropriate adult supervision strategies are needed to progress toward this goal. 

## Figures and Tables

**Table 1:** Baseline characteristics of children under five in the entire population.

Characteristics	Total (*n* = 112,664) *n* (%)	Injury Death (*n* = 126) *n* (%)	Alive (*n* = 112,538) *n* (%)	*p*-Value
**Supervision:**				<0.01
Yes	20,569 (18.3)	11 (8.7)	20,558 (18.3)	
No	92,095 (81.7)	115 (91.3)	91,980 (81.7)	
**Sex:**				0.6
Female	55,372 (49.1)	59 (46.8)	55,313 (49.2)	
Male	57,292 (50.9)	67 (53.2)	57,225 (50.8)	
**Age (years):**				<0.01
<1	22,141 (19.7)	7 (5.6)	22,134 (19.7)	
1–4	90,523 (80.3)	119 (94.4)	90,404 (80.3)	
**Socioeconomic index:**				0.9
Lowest	22,946 (20.4)	26 (20.6)	22,920 (20.4)	
Low	20,355 (18.0)	26 (20.6)	20,329 (18.0)	
Middle	22,413 (19.9)	24 (19.1)	22,389 (19.9)	
High	22,270 (19.8)	23 (18.3)	22,247 (19.8)	
Highest	24,680 (21.9)	27 (21.4)	24,653 (21.9)	

**Table 2:** Characteristics of children under five, cases and selected controls.

Characteristics	Total (*n* = 504) *n* (%)	Cases (*n* = 126) *n* (%)	Controls (*n* = 378) *n* (%)	Unadjusted MOR (95% CI)
**Supervision:**				
Yes	93 (18.4)	11 (8.7)	82 (21.7)	1.0
No	411 (81.6)	115 (91.3)	296 (78.3)	2.9 (1.5–5.7)
**Sex:**				
Female	253 (50.2)	59 (46.8)	194 (51.3)	1.0
Male	251 (49.8)	67 (53.2)	184 (48.7)	1.2 (0.8–1.8)
**Age (years):**				
<1	78 (15.5)	7 (5.6)	71 (18.8)	1.0
1–4	426 (84.5)	119 (94.4)	307 (81.2)	3.8 (1.7–8.5)
**Socioeconomic index:**				
Lowest	102 (20.2)	26 (20.6)	76 (20.1)	1.2 (0.6–2.3)
Low	105 (20.8)	26 (20.6)	79 (20.9)	1.1 (0.6–2.1)
Middle	106 (21.0)	24 (19.1)	82 (21.7)	1.0
High	98 (19.4)	23 (18.3)	75 (19.8)	1.05 (0.5–2.1)
Highest	93 (18.6)	27 (21.4)	66 (17.5)	1.5 (0.7–3.1)

**Table 3:** Characteristics of adult caregivers of selected cases and controls.

Characteristics	Total (*n* = 504) *n* (%)	Cases (*n* = 126) *n* (%)	Controls (*n* = 378) *n* (%)	Unadjusted MOR (95% CI)
**Age (years):**				
18–24	18 (3.6)	2 (1.6)	16 (4.2)	0.4 (0.1–1.7)
25–64	420 (83.3)	98 (77.8)	322 (85.2)	1.0
≥65	66 (13.1)	26 (20.6)	40 (10.6)	2.7 (1.4–5.1)
**Education:**				
None	181 (35.9)	49 (38.9)	132 (34.9)	1.0
Primary	179 (35.5)	49 (38.9)	130 (34.4)	0.9 (0.6–1.6)
Secondary	121 (24.0)	24 (19.0)	97 (25.7)	0.6 (0.3–1.3)
Advanced level and above	23 (4.6)	4 (3.2)	19 (5.0)	0.5 (0.1–1.6)
Occupation *:				
Trading	95 (18.9)	17 (13.5)	78 (20.6)	1.0
Agriculture/farming	144 (28.6)	35 (27.8)	109 (28.8)	1.5 (0.7–2.9)
Skilled labor	94 (18.7)	25 (19.8)	69 (18.3)	1.7 (0.8–3.4)
Unskilled labor	25 (5.0)	6 (4.8)	19 (5.0)	1.4 (0.4–4.3)
Transport worker	37 (7.3)	14 (11.1)	23 (6.1)	3.0 (1.2–7.2)
Student	2 (0.4)	1 (0.8)	1 (0.3)	4.4 (0.3–73.7)
Retired/Unemployed/Housewife	106 (21.0)	28 (22.2)	78 (20.6)	1.6 (0.8–3.4)
**Marital status:**				
Married	476 (94.4)	120 (95.2)	356 (94.2)	1.0
Never married/divorced/widowed	28 (5.6)	6 (4.8)	22 (5.8)	0.8 (0.3–2.1)

* Information on the caregivers’ occupation was missing for one control (total missing *n* = 1 (0.2%); control missing *n* = 1 (0.3%)).

**Table 4:** Association of unintentional injury-related mortality with adult caregiver supervision and other independent variables.

Characteristics	Unadjusted MOR (95% CI)	Adjusted MOR (95% CI)
**Supervision:**		
Yes	1.0	1.0
No	2.9 (1.5–5.7)	3.3 (1.6–7.0)
**Sex of the child:**		
Female	1.0	1.0
Male	1.2 (0.8–1.8)	1.1 (0.7–1.8)
**Age of the child (years):**		
<1	1.0	1.0
1–4	3.8 (1.7–8.5)	5.2 (2.2–12.2)
**Socioeconomic index:**		
Lowest	1.2 (0.6–2.3)	0.7 (0.3–1.5)
Low	1.1 (0.6–2.1)	0.8 (0.4–1.7)
Middle	1.0	1.0
High	1.05 (0.5–2.1)	0.8 (0.4–1.7)
Highest	1.5 (0.7–3.1)	1.2 (0.5–2.6)
**Caregiver’s age (years):**		
18–24	0.4 (0.1–1.7)	0.3 (0.03–2.2)
25–64	1.0	1.0
≥65	2.7 (1.4–5.1)	3.4 (1.5–7.6)
**Caregiver’s education:**		
None	1.0	1.0
Primary	0.9 (0.6–1.6)	0.9 (0.5–1.6)
Secondary	0.6 (0.3–1.3)	0.7 (0.3–1.4)
A levels and above	0.5 (0.1–1.6)	0.4 (0.1–1.5)
**Caregiver’s occupation:**		
Trading	1.0	1.0
Agriculture/farming	1.5 (0.7–2.9)	1.4 (0.6–3.0)
Skilled labor	1.7 (0.8–3.4)	2.0 (0.9–4.3)
Unskilled labor	1.4 (0.4–4.3)	1.9 (0.6–6.4)
Transport worker	3.0 (1.2–7.2)	3.4 (1.3–9.0)
Student	4.4 (0.3–73.7)	-
Retired/Unemployed/Housewife	1.6 (0.8–3.4)	1.4 (0.6–3.3)
**Caregiver’s marital status:**		
Married	1.0	1.0
Never married/divorced/widowed	0.8 (0.3–2.1)	0.4 (0.1–1.4)

## Supplementary Materials

The following are available online at www.mdpi.com/1660-4601/14/5/515/s1, Table S1: Comparison of proportions of independent variables between controls and alive children under five years of age in the source population, Table S2: Comparison of proportions of independent variables between controls and total children under five years age in the source population.

## Abbreviations

The following abbreviations are used in this manuscript:

CIPRB	The Center for Injury Prevention Research Bangladesh
95% CI	95% confidence interval
HICs	high income countries
ICDDR,B	International Center for Diarrheal Disease Research, Bangladesh
LMICs	Low- and middle-income countries
MOR	matched odds ratio
SOLID	Saving of Children’s Lives from Drowning
