# Supplementary material for: Caregiver Supervision Practices and Risk of Childhood Unintentional Injury Mortality in Bangladesh
Source: Int J Environ Res Public Health. 2017 May 11;14(5):515. doi: 10.3390/ijerph14050515 (PMC5451966; doi:10.3390/ijerph14050515)
Supplement: Supplementary file 1 [file ijerph-14-00515-s001.pdf]

Supplementary Materials: Caregiver Supervision Practices and Risk of Childhood Unintentional Injury Mortality in Bangladesh

Khaula Khatlani, Olakunle Alonge, Aminur Rahman, Dewan Md. Emdadul Hoque, Al-Amin Bhuiyan, Priyanka Agrawal and Fazlur Rahman

Table S1. Comparison of proportions of independent variables between controls and alive children under five years of age in the source population

Characteristics	Controls (n = 378) n (%)	Alive Children Under Five Years Age (n = 112,538) n (%)	p-Value
Supervision:			
Yes	82 (21.7)	20,558 (18.3)	0.08
No	296 (78.3)	91,980 (81.7)	0.09
Sex:			
Female	194 (51.3)	55,313 (49.2)	0.4
Male	184 (48.7)	57,225 (50.8)	0.4
Age (years):			
<1	71 (18.8)	22,134 (19.7)	0.7
1–4	307 (81.2)	90,404 (80.3)	0.7
Socioeconomic index:			
Lowest	76 (20.1)	22,920 (20.4)	0.9
Low	79 (20.9)	20,329 (18.0)	0.2
Middle	82 (21.7)	22,389 (19.9)	0.4
High	75 (19.8)	22,247 (19.8)	0.9
Highest	66 (17.5)	24,653 (21.9)	0.03

Table S2. Comparison of proportions of independent variables between controls and total children under five years age in the source population

Characteristics	Controls (n = 378) n (%)	Total Children Under Five Years Age (n = 112,664) n (%)	p-Value
Supervision:			
Yes	82 (21.7)	20,569 (18.3)	0.09
No	296 (78.3)	92,095 (81.7)	0.09
Sex:			
Female	194 (51.3)	55,372 (49.1)	0.4
Male	184 (48.7)	57,292 (50.9)	0.4
Age (years):			
<1	71 (18.8)	22,141 (19.7)	0.7
1–4	307 (81.2)	90,523 (80.3)	0.7
Socioeconomic index:			
Lowest	76 (20.1)	22,946 (20.4)	0.9
Low	79 (20.9)	20,355 (18.0)	0.2
Middle	82 (21.7)	22,413 (19.9)	0.4
High	75 (19.8)	22,270 (19.8)	1.0
Highest	66 (17.5)	24,680 (21.9)	0.03

© 2017 by the authors; licensee MDPI, Basel, Switzerland. This article is an open access article distributed under the terms and conditions of the Creative Commons by Attribution (CC-BY) license (http://creativecommons.org/licenses/by/4.0/).
